# Increased ultrasensitive C-reactive protein is not associated with obesity in hospitalized heart failure patients

**DOI:** 10.1590/S1679-45082016AO3705

**Published:** 2016

**Authors:** Vânia Ames Schommer, Airton Tetelbom Stein, Aline Marcadenti, Estefania Inez Wittke, André Luís Câmara Galvão, Guido Bernardo Aranha Rosito

**Affiliations:** 1 Universidade Federal de Ciências da Saúde de Porto Alegre, Porto Alegre, RS, Brazil; 2 Hospital Nossa Senhora da Conceição, Porto Alegre, RS, Brazil

**Keywords:** Heart failure, Obesity, C-reactive protein

## Abstract

**Objective:**

To evaluate the association between obesity and levels of high-sensitivity C-reactive protein (hs-CRP) in patients with heart failure admitted to a tertiary hospital.

**Methods:**

Cross-sectional study with a consecutive sampling of hospitalized patients with heart failure. Sociodemographic and clinical data were collected, and the nutritional status was assessed through indicators such as body mass index (in kg/m^2^), waist circumference (in cm), waist-hip ratio, triceps skinfold (in mm) and subscapularis skinfold (in mm). Neck circumference (in cm) was measured as well as serum levels of hs-CRP, in mg/L.

**Results:**

Among 123 patients, the mean age was 61.9±12.3 years and 60.2% were male. The median of hs-CRP was 8.87mg/L (3.34 to 20.01). A tendency to an inverse correlation between neck circumference and hs-CRP was detected (r=-0.167; p=0.069). In the multiple linear regression analysis, after adjustment for age, disease severity (NYHA classification III and IV, low ejection fraction, left ventricular dysfunction during diastole), and infectious conditions there was an inverse association between hs-CRP and neck circumference (ß=-0.196; p=0.03) and subscapularis skinfold (ß=-0.005; p=0.01) in the total sample, which was not maintained after the stratification by sex.

**Conclusion:**

Increased levels of hs-CRP in patients hospitalized for heart failure were not associated with obesity.

## INTRODUCTION

Heart failure (HF) is characterized as a highly complex syndrome due to the structural and functional alterations of the heart, involving several causal and compensatory mechanisms.^(1)^ This is a condition considered endemic in developing countries, such as Brazil, and it is a frequent cause of death and admissions to hospital.^(2)^

Among the possible factors associated with the genesis of H F, obesity stands out.^(3)^ Nevertheless, it is suggested that patients diagnosed with HF and with excess weight have greater survival when compared to normal-weight patients diagnosed with the disease, as per the body mass index (BMI).^(4)^ The adipose tissue, present in an excessive amount in obese individuals, is an endocrine organ, which is involved in the regulation of physiological and pathological mechanisms (including inflammatory processes),^(5)^ in which acute phase protein concentrations, as well as that of pro-inflammatory cytokines, are elevated.^(6)^

C-reactive protein CRP is an amply studied acute phase inflammatory mediator of cardiovascular diseases^(7)^ and is considered an unspecific marker of systemic inflammation.^(8)^ High-sensitivity CRP (hs-CRP) has prognostic value for ischemic cardiopathy and H F.^(9,10)^ High levels of hs-CRP in patients with HF are associated with increased morbidity and mortality in cases of ischemic and non-ischemic etiology.^(11)^

Both neurohumoral and inflammatory activations are considered important mechanisms for the progression of HF. Patients with HF and high levels of hs-CRP were twice as likely to be readmitted to hospital and die, when compared to individuals with lower levels of hs-CRP.^(11)^

## OBJECTIVE

To evaluate the association between central and general obesity indicators and levels of high-sensitivity C-reactive protein among patients with heart failure admitted to a tertiary care hospital.

## METHODS

This is a cross-sectional analysis of the baseline of a cohort study which consecutively enrolled patients admitted due to HF at the Cardiology Service of the *Hospital Nossa Senhora da Conceição* (HNSC) in the city of Porto Alegre (RS).

The inclusion criteria were New York Heart Association (NYHA) class I-IV history of HF;^(12)^ systolic and diastolic HF; age between 20 and 85 years; absence of history or clinical evidence of severe complications related to HF over the previous 30 days; patients who resided in the metropolitan region of Porto Alegre, and who agreed to participate in the study. The exclusion criteria were situations that precluded anthropometric evaluation (amputation of limbs or sequelae from a stroke), and unwillingness to participate in the study.

The initial clinical evaluation was done by physicians and medical students trained and with the supervision of cardiologists. Training was given during the pilot study, in which the students applied the questionnaire to hospitalized patients, with characteristics similar to those who were, in fact, enrolled in the study.

The variables studied consisted of clinical and sociodemographic data and issues relative to smoking and alcohol consumption, besides anthropometric evaluations done by equally trained dieticians and nutrition students. Associated morbidities were identified from the patient's past history; prior medical diagnoses were recorded: type 2 *diabetes mellitus,* systemic arterial hypertension, chronic renal failure, anemia, and dyslipidemia.

Data on inflammatory conditions among the patients were collected by a physician from the organization, considering the following inflammatory diseases: chronic obstructive pulmonary disease exacerbated during hospitalization, pneumonia, rheumatic diseases, autoimmune diseases, urinary tract disease, and sepsis. The levels of hs-CRP were evaluated according to the protocol of the HNSC clinical analyses laboratory, based on the nephelometry method.^(13)^

Weight was checked by means of a digital scale, with the patient wearing light clothing and no shoes. In cases of volemic alterations confirmed by means of a physical examination (presence of edema), the patient's weight was verified after treatment for this condition, and if there was residual edema, a formula for an estimate of body weight adjusted for edema was used.^(14)^ Height was checked with the help of a fixed stadiometer and the BMI was calculated; for classification of overweight and obese, the cutoff points were ≥25kg/m^2^ and ≥30kg/m^2^, respectively.^(15)^

With an inelastic tape measure, the following measurements in centimeters were made: waist circumference (WC), considering the minimum circumference between the ribs and the pelvis; hip circumference (HC), with the maximum circumference in the hip region; and neck circumference (NC), at the midpoint of the neck. Abdominal obesity was defined as a value of WC ≥102cm for men and ≥88cm for women,^(16)^ and the waist-hip ratio (WHR) >0.95 for men and >0.85 for women.^(17)^

Checking of the tricipital skin fold (TSF) was done with the patient standing or sitting, with the non-dominant arm freely extended along the body; the measurement was checked at the midpoint between the acromial process of the scapula and the olecranon of the ulna. To measure the subscapular skin fold (SSF), the point below the inferior angle of the scapula was identified, with the shoulder and arm relaxed, parallel to the natural cleavage lines of the skin.^(18)^ The skin folds were identified by means of an adipometer.

HF etiology was defined by the cardiologist and collected in the patient medical record (secondary data). The patients underwent transthoracic echocardiography with color Doppler, and measures of systolic function and calculation of the ejection fraction (EF) were made using Teichholz formula;^(19)^ individuals with segment alterations in left ventricle contractility had their EF determined by Simpson method. All patients did the stress test on a treadmill with a slope protocol.^(20)^

The study was approved by the Research Ethics Committees of the HNSC, under protocol 10–118, of August 2010, and of the *Universidade Federal de Ciências da Saúde de Porto Alegre* (UFCSPA), under protocol 1,747, of June 2012. All individuals participating in the study signed the Informed Consent Form.

The sample size was calculated by means of the WinPepi program, version 11.15. Considering a prevalence of obesity of 30% among patients with HF (ratio of 2:1), a prevalence of elevated hs-CRP of 60% among obese (exposed), and of 30% among the non-obese, the size of the sample was estimated as at least 96 patients, for a 95% confidence level and 80% power.^(21,22)^

The statistical analysis was made using the Statistical Package for the Social Sciences (SPSS), version 22.0. Continuous variables were described by means and standard deviation when they showed a symmetric distribution, and by medians and interquartile interval in asymmetric distributions. To evaluate the difference in the continuous symmetric variables, Student's *t* test was used; for asymmetric variables, Mann-Whitney's test was employed; and for categorical variables, Fisher's exact test. To evaluate correlations, Pearson's correlation was used, and to adjust confounding variables, multiple linear regression was employed. In all analyses, a 5% level of significance was considered.

## RESULTS

A total of 123 inpatients were evaluated predominantly presenting acute decompensation of HF, with a mean age of 61.9±12.3 years, 60.2% males, and 84.3% of them classified as NYHA functional classes III and IV. Table 1 presents the general characteristics of the sample.

**Table 1:** General characteristic of the population

		Total	Male (n=74)	Female (n=49)	p value
Age (years)		61.97±12.29	61.30±11.62	62.98±13.29	0.46*
Self-referred ethnicity					
	White	89 (72.4)	53 (71.6)	36 (73.5)	0.25†
	Mixed/mulatto	16 (13)	7 (9.5)	9 (18.4)	
	Black	13 (10.6)	10 (13.5)	3 (6.1)	
	Other	5 (4)	4 (2.96)	1 (2)	
Smoking					
	Smoker	15 (12.3)	11 (15.1)	4 (8.2)	0.16†
	Former smoker	59 (48.4)	38 (52.1)	21 (42.9)	
	Never smoked	48 (39.3)	24 (32.9)	24 (49)	
NYHA functional class#					
	I	2 (1.7)	1 (1.4)	1 (2.1)	0.23†
	II	17 (14)	7 (9.6)	10 (20.8)	
	III	66 (54.5)	44 (60.3)	22 (45.8)	
	IV	36 (29.8)	21 (28.7)	15 (31.3)	
	EF (%)	39.92±14.45	36.45±13.44	45.19±14.46	0.001*
HF etiology					
	SAH	42 (34.1)	19 (25.7)	23 (46.9)	0.10†
	Cardiomyopathy	39 (31.7)	29 (39.2)	10 (20.4)	
	Ischemic disease	29 (23.6)	18 (24.3)	11 (22.4)	
	Other	13 (10.5)	8 (10.8)	5 (10.2)	
Associated morbidities					
	SAH	97 (79.5)	54 (73)	43 (89.6)	0.38†
	Dyslipidemia	45 (36.9)	25 (33.8)	20 (41.7)	0.44†
	DM2	42 (34.4)	23 (31.1)	19 (39.6)	0.43†
	CRF	25 (20.5)	14 (18.9)	11 (22.9)	0.64†
	Anemia	21 (17.2)	4 (5.4)	17 (35.4)	<0.001†
Blood pressure					
	SBP (mm/Hg)	122.66±18.55	120.89±18.64	125.39±18.26	0.20*
	DBP (mm/Hg)	75.38±11.49	74.58±11.78	76.61±11.04	0.35*
	hs-CRP (mg/L)‡	8.87 (0.79–180)	11 (0.8–101)	7.80 (0.9–180)	0.23§

Categorical variables expressed in n (%) and continuous variables as mean ±standard deviation or median (interquartile interval).

* Student's t test;

† Fisher's exact test;

‡ Evaluated in 119 individuals;

§ Mann-Whitney's test;

# Evaluated in 121 individuals.

NYHA: New York Heart Association; EF: ejection fraction; HF: heart failure; SAH: systemic arterial hypertension; DM2: type 2 *diabetes mellitus*; CRF: chronic renal failure; SBP: systolic blood pressure; DBP: diastolic blood pressure; hs-CRP: high-sensitivity C-reactive protein.

As to associated morbidities, 79.5% of patients presented with a past history of systemic arterial hypertension, 36.9% of dyslipidemia, 32% of type 2 *diabetes mellitus*, 20.5% of chronic renal failure, and 17.2% of anemia. There was no significant difference between the morbidities according to sex, with exception of the prevalence of anemia, statistically higher among women (35.4% *versus* 5.4%; p<0.001). The mean EF was 39.9±14.5%, and women showed a higher EF compared to men: 45.2±14.5% and 36.5±13.4%, respectively (p=0.001).

**Figure 1:**
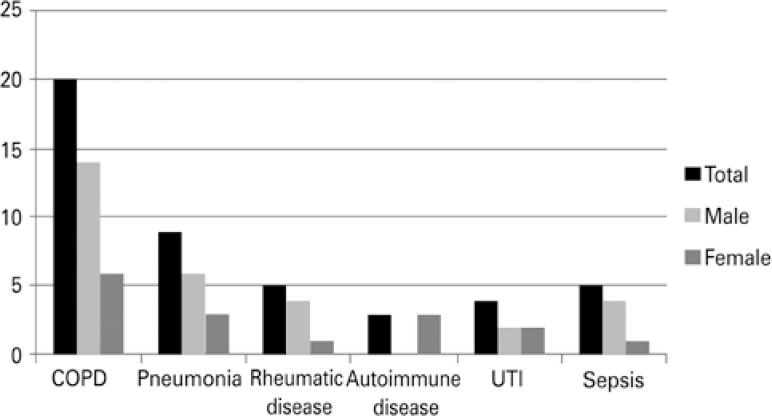
Distribution of infectious conditions in the sample CPOD: chronic pulmonary obstructive disease; UTI: urinary tract infection.

Thirty-four individuals were identified (28.5%) with some type of infection concomitant with HF during hospitalization. Figure 1 shows the percentage of individuals identified in each clinical picture according to sex, and there was no significant difference between men and women.

The mean BMI was 28.5±6.5kg/m^2^, with a prevalence of excess weight in the sample of 64.2%. Obesity was more frequent in women (46.9%), and overweight in men (39.2%). Table 2 displays the classifications of obesity and overweight according to different criteria of anthropometric evaluation per sex.

**Table 2:** Classification of obesity, as per different criteria, according to sex

	Overweight			Obesity		
	Total	Male (n=74)	Female (n=49)	Total	Male (n=74)	Female (n=49)
BMI (kg/m^2^)*	38 (30.9)	29 (39.2)	9 (18.4)	41 (33.3)	18 (24.3)	23 (46.9)
TSF (mm)†	6 (4.9)	1 (1.4)	5 (10.2)	74 (60.2)	58 (78.4)	16 (32.7)
WC (cm)	–	–	–	66 (53.7)	26 (35.1)	40 (81.6)
WHR	–	–	–	98 (79.7)	57 (77)	41 (83.7)

* According to the World Health Organization, 2004;

† according to Blackburn, 1979.

BMI: body mass index; TSF: tricipital skinfold; WC: waist circumference; WHR: waist-hip ratio.

For hs-CRP analyses, data in reference to 119 patients were used due to baseline losses, because the patient was discharged from the hospital before the test was requested. The median hs-CRP in the sample was 8.87mg/L (3.34 a 20.01), and table 3 represents the distribution of the nutritional status identified by different criteria, as per the hs-CRP tertiles.

**Table 3:** Tertiles of high-sensitivity C-reactive protein and distribution of the nutritional status as per anthropometric variables

		Tertiles of hs-CRP		
BMI (kg/m^2^)		1 ≤5.5 (mg/L)	5.6 - 16 (mg/L)	≥16.1 (mg/L)
	Malnutrition	0 (0)	1 (1.19)	0 (0)
	Normal weight	17 (20.23)	12 (14.28)	13 (15.47)
	Overweight	12 (14.28)	12 (14.28)	12 (14.28)
	Obesity	11 (13.09)	15 (17.85)	14 (16.66)
TSF (mm)				
	Malnutrition	15 (17.85)	11 (13.09)	7 (8.33)
	Normal weight	1 (1.19)	2 (2.39)	5 (5.95)
	Overweight	1 (1.19)	3 (3.57)	2 (2.38)
	Obesity	23 (27.37)	24 (28.56)	25 (29.75)
WC (cm)				
	Normal weight	19 (22.61)	15 (22.61)	19 (22.61)
	Obesity	21 (24.99)	25 (29.75)	20 (23.80)
WHR				
	Normal weight	9 (10.71)	5 (5.95)	9 (10.71)
	Obesity	31 (36.89)	35 (41.65)	30 (35.70)

Data expressed in n (%).

hs-CRP: high-sensitivity C-reactive protein; BMI: body mass index; TSF: tricipital skinfold; WC: waist circumference; WHR: waist-hip ratio.

No statistically significant correlations were noted between hs-CRP levels and weight (r=-0.031; p=0.74), BMI (r=0.44; p=0.63), WC (r=-0.04; p=0.67), TSF (r=0.31; p=0.74), and SSF (r=0.02; p=0.86). However, a tendency was detected towards an inverse correlation between NC and hs-CRP (r=-0.167; p=0.069).

Multiple linear regression analysis, adjusted for age, severity of disease (NYHA classification III and IV, low EF, HF with preserved EF), and presence of infection indicated an inverse association relative to hs-CRP and NC (β=−0.196; p=0.03) and PCS (β=−0.005; p=0.01). However, after stratification for sex, only the tendency towards association for NC was maintained both in men (β=−0.23; p=0.07) and in women (β=−0.27; p=0.08) (Table 4).

**Table 4:** Multiple linear regression among high-sensitivity C-reactive protein levels and different anthropometric variables

Variable	Total		Male (n=74)		Female (n=49)	
	B	p value	B	p value	B	p value
Weight (kg)	−0.096	0.341	−0.002	0.986	−0.231	0.211
Body mass index (kg/m^2^)	−0.042	0.691	0.003	0.981	−0.100	0.578
Waist circumference (cm)	−0.054	0.592	−0.006	0.965	−0.113	0.527
Neck circumference (cm)	−0.196	0.036	−0.231	0.073	−0.274	0.082
Tricipital skinfold (mm)	−0.020	0.844	−0.041	0.758	−0.58	0.774
Subscapular skinfold (mm)	−0.005	0.011	0.017	0.117	−0.156	0.525
Waist-hip ratio	−1.255	1.465	−0.026	0.836	−0.109	0.486

## DISCUSSION

The evidence of a linear relation between obesity and hs-CRP found in healthy people does not seem to occur in HF patients. In our analysis, on the contrary, there was an inverse association between some anthropometric variables and hs-CRP levels.

Obesity is a condition of chronic inflammation, mediated by an increased production of cytokines and hs-CRP by adipocytes.^(23)^ There is an association between hs-CRP and the anthropometric indicators in patients with no history of HF.^(24–26)^ Sanip et al., in a study with 91 healthy postmenopausal women, found a correlation between hs-CRP and obesity, highlighting BMI (r=0.281; p=0.007), WC (r=0.340; p=0.001), and HC (r=0.257; p=0.014).^(24)^

In our study, it was possible to observe high levels of hs-CRP, a fact that likely can be explained by severity of disease, exacerbation of HF symptoms, or presence of comorbidities that led the patients to hospitalization. The high level of hs-CRP found corroborates the findings of Chen et al., in which patients evaluated during hospital admission presented with mean hs-CRP of 53±57.7mg/L.^(27)^ On the other hand, among Japanese assessed as outpatients (mean hs-CRP of 10.9±18mg/L as baseline), the individuals classified in the largest quartile of hs-CRP (>11mg/L) had the worst prognosis after three years.^(28)^

The sample distribution was homogeneous relative to sex; 60.2% of individuals were men, thus allowing extrapolation of results in a more significant manner for both sexes. These findings were different from those of most observational studies, which address the paradox of obesity, when the difference between sexes tends towards an absolute majority of men.^(29,30)^

Most of the sample was classified as severe, as per the NYHA functional classification, differing from the results of a cohort carried out in Porto Alegre, in which only 17% of patients were in functional classes III and IV.^(4)^ This difference can be justified by the site of data collection, that is, the present study was performed during hospitalization, whereas the comparative cohort was carried out with outpatients, in whom likely patients of less gravity are seen.

The mean EF identified was similar to that found in other studies.^(27,31)^ Galvao et al. identified differences between sexes as to EF similarly to our findings, in the study analysis from the Acute Decompensated Heart Failure National Registry (ADHERE),^(32)^ considering that these differences can be justified by the better prognosis that women showed in HF progression compared to men.

In our study, we identified a prevalence of excess weight evaluated by means of a BMI of 64.2%, in which 30.9% were overweight and 33.3% were obese. Clark et al. described that, among individuals with HF, the prevalence of overweight varies between 31 and 40%, and from 32 to 49% for obesity.^(29)^ These data corroborate those of other studies, displaying a similar prevalence of overweight and obesity.^(33–36)^

The mean NC identified was similar to that observed in another study conducted among individuals with HF and sleep apnea,^(37)^ and the mean TSF was superior to that observed in outpatients.^(4)^ As to the prevalence of overweight and obesity classified according to TSF, the present study identified 4.9% and 60.2%, respectively; Casas-Vara et al. found similar data relative to overweight, but great discrepancy in the prevalence of obesity (14.2% according to this classification criterion).^(33)^ As to SSF, we identified means similar to those of other authors.^(38)^

There are few studies evaluating the association of anthropometric measurements and the inflammatory profile of HF patients. In patients hospitalized for HF and evaluated in our study, the excess weight did not lead to increased hs-CRP. It is possible that the increase in weight be a benefit to these individuals, due to greater energy reserve, contrary to cardiac cachexia.^(39)^ Another hypothesis is that HF causes ischemia and intestinal edema, allowing bacterial translocation and favoring the formation of endotoxins, contributing to an inflammatory state in individuals with HF, regardless of the degree of excess weight, favoring the loss of muscle mass, but not necessarily of fat mass.^(40)^

Some limitations must be considered: the study design (cross-sectional), which does not allow a definition of causality; and the sample size hindered the subgroup analysis. Another point to be highlighted refers to severity of patients enrolled for the sample: one cannot discard the fact that there is an association between hs-CRP and obesity in individuals with milder degrees of HF, making it difficult to generalize our results for patients with HF and less severe conditions. We also point out that, in the mathematical formula for estimating body weight adjusted for edema, despite being amply used in clinical practice, a relatively subjective approach is used. Additionally, the techniques used to identify skinfolds by means of adipometer do not have the same accuracy of the imaging methods to identify the body composition; and in cases of residual edema that is still present, these folds might have been overestimated.

## CONCLUSION

In patients hospitalized due to heart failure, there is a significant increase in high-sensitivity C-reactive protein, but no association was found with obesity. Although obesity contributes towards high concentrations of high-sensitivity C-reactive protein in individuals with normal heart function, the exacerbated inflammatory state observed in severe cardiac failure seems to be a factor that influences these concentrations more significantly. Other studies that evaluate the prognostic impact of high-sensitivity C-reactive protein according to the nutritional status should be conducted to better utilize inflammatory markers in heart failure patients.
